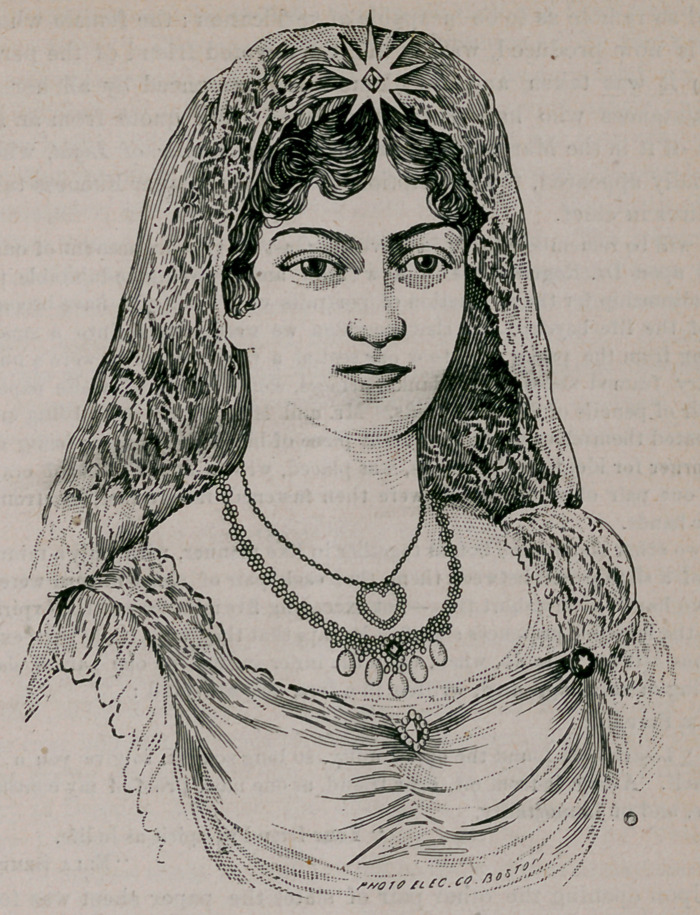# Spirit Likeness

**Published:** 1887-09

**Authors:** 


The above cut represents a portrait in crayon, made by invisible agencies, through the instrumentality of Doctor and Mrs. Henry Rogers, two very remarkable sensitives for psychic manifestations of this wonderful phaze.

The readers of the JOURNAL have been made somewhat familiar with the *modus operandi* respecting the production of these marvels of art, by the presentation, in our May number, of the likeness of an ancient Egyptian, with an account in detail of the manner of its production. The two likenesses were taken under circumstances so nearly alike, that a description of the details of the one is a description of the other.

There is, however, this difference between the two. Whilst the male likeness "Amarona," published by us in May, is that of an ancient, of a period so remote as to be incapable of verification ; the female whose likeness is now produced, was, in life, the devoted friend of the person for whom it was taken, and the likeuess is pronounced by all her former acquaintances who have seen it, *faultless.* We quote from an account given of it in the March 26th number of the *Banner of Light*, wherein it originally appeared, the description of another smaller likeness taken for its editor in chief.

"It will be remembered that in November last we gave an account of our having called upon Dr. Rogers while in New York, and receiving indubitable proof of his mediumship for the production of portraits of friends who have become denizens of the life beyond. On that occasion we were invited into a small room opening from the parlor, and took our seat at a table on which were a number of ordinary framed slates, with blank surfaces, some sheets of white paper and a number of pencils of different kinds. Mr. and Mrs. Rogers, both being mediums, also seated themselves at the table. A piece of blank paper, after being depleted of a corner for identification by us, was placed, with a small piece of crayon, between one pair of slates, which were then fastened, face to face, by strong india-rubber bands.

"Two other slates were bound together in like manner, with only a minute fragment of a slate pencil between them, then each pair of slates in turn were held in our own hands a very short time—not exceeding five minutes—at the expiration of which the invisible presences signified by raps that they were ready to be examined. This was left for us to do, when, upon the inner surface of one pair of slates the following message appeared, written in a free, delicate hand :

"DEAR FRIEND :

"I have here found the opportunity, so long sought, to give you a portrait of myself. Accept it from me, dear friend, as one more proof of my continued existence, and of immortality.

"Your friend in spirit as in life,

"ELLA SEMPSON."

"Upon opening the other pair of slates the paper sheet was found to contain a beautifully executed crayon drawing (head and bust) of a young lady. The likeness was by us recognized as that of the young friend whose message we have given above, and certainly, as a work of art, in linear drawing and shading it is faultless."

These are truths, which are every day being brought home to the denizens of earth, in such overwhelming numbers, that it is impossible to resist them, and why should we wish to resist them ?

## Figures and Tables

**Figure f1:**